# HIF‐1α inhibition alleviates the exaggerated exercise pressor reflex in rats with peripheral artery disease induced by femoral artery occlusion

**DOI:** 10.14814/phy2.14676

**Published:** 2020-12-23

**Authors:** Lu Qin, Jianhua Li

**Affiliations:** ^1^ Heart and Vascular Institute The Pennsylvania State University College of Medicine Hershey PA USA

**Keywords:** exercise pressor reflex, HIF‐1α, muscle afferents, peripheral artery disease

## Abstract

Hypoxia‐inducible factor 1α (HIF‐1α) is a transcription factor mediating adaptive responses to hypoxia and ischemia. Our previous work showed that HIF‐1α is increased in muscle sensory nerves of rats with peripheral artery disease (PAD) induced by femoral artery occlusion. The present study was further to examine the role played by HIF‐1α in regulating the response of arterial blood pressure (BP) to the activation of muscle afferent nerve during static muscle contraction in rats with femoral artery occlusion. A rat model of femoral artery ligation was used to study PAD in this study. Western blot analysis was employed to examine the protein levels of HIF‐1α in the dorsal root ganglion (DRG) tissues. BAY87, a synthesized compound with the characteristics of highly potent and specific suppressive effects on expression and activity of HIF‐1α, was given into the arterial blood supply of the ischemic hindlimb muscles before the exercise pressor reflex was evoked by static muscle contraction. First, HIF‐1α was increased in the DRG of occluded limbs (optical density: 0.89 ± 0.13 in control versus 1.5 ± 0.05 in occlusion; *p* < 0.05, n = 6 in each group). Arterial injection of BAY87 (0.2 mg/kg) then inhibited expression of HIF‐1α in the DRG of occluded limbs 3 hr following its injection (optical density: 1.02 ± 0.09 in occluded limbs with BAY87 versus 1.06 ± 0.1 in control limbs; *p* > 0.05, n = 5 in each group). In addition, muscle contraction evoked a greater increase in BP in occluded rats. BAY87 attenuated the enhanced BP response in occluded rats to a greater degree than in control rats. Our data suggest that the inhibition of HIF‐1α alleviates the exaggeration of the exercise pressor reflex in rats under ischemic circumstances of the hindlimbs in PAD induced by femoral artery occlusion.

## 1 INTRODUCTION

During exercise, contracting skeletal muscle stimulates thin fiber afferent nerves (group III &IV) innervating muscles and this elicits a reflex increase in sympathetic nerve activity (SNA), an effect which in turn increases arterial blood pressure (BP), heart rate (HR), myocardial contractility and peripheral vascular resistance, a mechanism termed the exercise pressor reflex (Mitchell et al., 1983). In peripheral artery disease (PAD), systolic and diastolic BP rise significantly in the patients with PAD than in the normal subjects during walking (Baccelli et al., 1997). Notably, the exercise pressor reflex plays a crucial role in evoking the exaggerated BP response to walking in PAD patients (Baccelli et al., 1999). Using a rat model of femoral artery ligation to study PAD in humans (Waters et al., 2004), prior studies also demonstrated that the SNA and BP responses to static muscle contraction and stimulation of metabolic receptors in muscle afferent nerves are amplified in occluded rats as compared with control rats (Li & Xing, 2012).

Critical limb ischemia frequently appears in PAD due to atherosclerosis (Garcia, 2006; Nehler et al., 2003). In general, insufficient blood flow to metabolic demands of tissue leads to impairments in physiological functions (Dormandy et al., 1999). Particularly, the consumption of oxygen is lower in PAD patients than in health controls (Hiatt et al., 1987). The most common symptom is intermittent claudication, which is worsened by intense exercise due to muscle ischemia but subsides at rest when the metabolic demand of the muscles becomes low (Rejeski et al., 2008).

Hypoxia‐inducible factor‐1 (HIF‐1) is a heterodimeric protein composed of constitutively expressed HIF‐1α and HIF‐1β subunits (Wang et al., 1995). In the two subunits, oxygen‐sensitive HIF‐1α accumulates rapidly under hypoxic conditions and modulates the expression of several target genes in protecting tissues against ischemia and infarction (Iyer et al., 1998; Manalo et al., 2005). Thus, HIF‐1α is considered as a transcription factor mediating adaptive responses to hypoxia and ischemia (Iyer et al., 1998; Manalo et al., 2005). The abnormalities in HIF‐1 were observed in exercising the skeletal muscle of PAD (Gao et al., 2012). In our previous study (Gao & Li, 2013), the role played by HIF‐1α in modulating the exercise pressor reflex was examined in rats with femoral artery occlusion, showing that HIF‐1α was increased in the dorsal root ganglion (DRG) after femoral artery occlusion. It was also observed that dimethyloxalylglycine (DMOG), a competitive inhibitor of HIF‐α prolyl hydroxylase, maintained HIF‐1α levels in the DRG after it was given into the hindlimb muscles (Gao & Li, 2013). Nevertheless, the pressor response evoked by static muscle contraction was not significantly altered by DMOG in this previous study, indicating that femoral occlusion increases HIF‐1α in the DRG, but supplementary HIF‐1α per se is unlikely to alter the exercise pressor reflex.

In contrast, BAY 87‐2243 (BAY87) is a synthesized compound with characteristic of highly potent and specific suppressive effects on expression and activity of HIF‐1 observed in vitro and in vivo studies (Dilly et al., 2016; Ellinghaus et al., 2013; Gortz et al., 2016). BAY87 inhibits HIF‐1 target gene expression in hypoxic cells (Ellinghaus et al., 2013). It can specifically suppress the accumulation of HIF‐1α protein and expression of HIF‐1α target genes under hypoxia conditions by means of blocking mitochondria complex I activity through HIF‐mediated pathway (Dilly et al., 2016; Gortz et al., 2016). Thus, in this report, we examined if the activation of the exercise pressor reflex by muscle contraction was altered in control rats and occluded rats after the inhibition of HIF‐1α by the injection of BAY87 into the arterial blood supply of the hindlimb muscles. We hypothesized that BAY87 attenuates the amplification of HIF‐1α in the DRG of occluded rats thereby alleviating the exaggerated exercise pressor reflex.

## 2 METHODS

All animal experimental procedures were approved by *the Institutional Animal Care and Use Committee* of Penn State College of Medicine and complied with the National Institutes of Health (NIH) guidelines. A total number of sixty‐five experimental animals (male Sprague Dawley rats, 4–6 weeks old) were obtained from Charles River Laboratory and housed in individual cages with free access to food and water and they were kept in a temperature‐controlled room (25°C) on a 12‐hr/12‐hr light/dark cycle.

### 2.1 Ligation of the femoral artery

Male Sprague Dawley rats (4–6 weeks old) were anesthetized with an isoflurane‐oxygen mixture (2–5% isoflurane in 100% oxygen). Then, the femoral artery on one limb was surgically exposed, dissected, and ligated ~3 mm distal to the inguinal ligament as previously described (Gao & Li, 2013). In control, the same procedures were performed on the other limb except that a suture was placed below the femoral artery but was not tied. For the experiments using the western blotting analysis, the limb in which the femoral artery was ligated served as “occluded limbs”; and the other limb served as “control limbs.” For the experiment of BP recording, the rats were divided between those that had the femoral artery ligation (as “occluded rats”) and those that had sham surgeries on the limb (as “control rats”). For post‐surgery pain relief and care, buprenorphine hydrochloride (0.05 mg/kg, subcutaneously) was administered before the surgery. Following the surgery, the animals were kept in the surgery room for 2–3 hr for post‐surgery observation, and then returned to the animal facility. Seventy‐two hours were then allowed for recovery before the reflex experiments began.

### 2.2 Western blot analysis

Six rats were used to examine the expression of HIF‐1α in the L4‐6 DRGs of control limbs and limbs after 72 hr of femoral occlusion. Nine rats were used to examine HIF‐1α in the L4‐6 DRGs at 30 min, 1 hr, and 3 hr (n = 3 in each group) after the injection of BAY87 (0.2 mg/kg; Xcess Biosci Inc) via Polyethylene (PE‐10) catheter inserted in the femoral artery. To conduct the femoral artery cannulation, a small incision was made in the femoral artery of the control rats to insert a catheter. In the PAD rats, the incision was made in the femoral artery distal to the previously occluded site. The catheter was then inserted into the artery toward the distal end to deliver the drug into the experimented limb. Meanwhile, the limbs of five rats with 72 hr of femoral occlusion were also injected with BAY87 (0.2 mg/kg) and HIF‐1α in the L4‐6 DRGs was examined at 3 hr after the injection. Western blot methods were performed as previously described (Gao & Li, 2013). In brief, DRGs of the rats were removed. All DRGs tissues from individual rats were sampled for western blot analysis. Total protein was then extracted by homogenizing DRG samples and the lysates were centrifuged. The supernatants were then collected for measurements of protein concentrations.

After being denatured by heating at 95°C for 5 min in an SDS sample buffer, the supernatant samples containing 20 μg of protein were loaded onto 4–20% Mini‐Protean TGX Precast gels and then electrically transferred to a polyvinylidene fluoride membrane. The membrane was blocked by 5% nonfat milk in 0.1% Tween‐TBS buffer and was then incubated overnight with primary antibody: rabbit anit‐HIF‐1α (1:500; Santa Cruz).

After being fully washed, the membrane was incubated with horseradish peroxidase‐linked anti‐rabbit secondary antibody (1:1000; Abcam) and visualized for immunoreactivity using an enhanced chemiluminescence system. The membrane was stripped and incubated with anti‐β‐actin (Sigma‐Aldrich) to show equal loading of the protein in the western blot analysis. The densities of HIF‐1α and β‐actin bands were determined using the NIH Scion Image Software. The expression of HIF‐1α protein was calculated by the optical density of the immunoreactive band/β‐actin band from the same lane. The value of optical density for each sample was then normalized to that of one of the control samples in the respective gel. The values of the averaged optical density for each group were determined by dividing the total accumulated normalized value by the sample size.

### 2.3 Examination of the exercise pressor reflex

The effect of the BAY‐87 on the exercise pressor reflex of control and occluded rats were examined by three parts of experiments: (a) the blood pressure response following the static muscle contraction; (b) the dosage‐effect of BAY‐87 (saline, 0.1 mg/kg, 0.2 mg/kg) on blood pressure response following static muscle contraction; and (c) the time‐course effect of BAY‐87 (0, 30 min, 60 min, 90 min, 2 hr, 3 hr, 3.5 hr, 4 hr, 4.5 hr after the injection of 0.2 mg/kg BAY‐87) on the blood pressure response following the static muscle contraction.

In general, the rats were anesthetized by the inhalation of an isoflurane oxygen mixture and an endotracheal tube was inserted and attached to a ventilator. Polyethylene (PE‐50) catheters were inserted into an external jugular vein and the right carotid artery for saline injection and measurement of BP. PE‐10 catheters were inserted into the femoral arteries for the injection of drugs (Saline or BAY‐87) into the arterial blood supply of the hindlimb muscles. The skin covering the hindlimb muscles was surgically separated from the muscle below to eliminate inputs from cutaneous afferents in the hindlimb. During the experiment, end‐tidal CO_2_, BP, and body temperature were monitored and maintained within normal ranges (Gao & Li, 2013; Xing et al., 2015). BP was measured by connecting the carotid arterial catheter to a pressure transducer. Mean arterial pressure (MAP) was obtained by integrating the arterial signal with a time constant of 4 s. HR was determined from the arterial pressure pulse.

The exercise pressor reflex was evoked by the static muscle contraction. For the preparation of the stimulated muscle contraction, a laminectomy was firstly performed to expose the lower lumbar and upper sacral portions of the spinal cord after the rats were placed in a spinal unit. The spinal roots were exposed and the right L4&5 ventral roots were visually identified with the assistance of an anatomical microscope. The peripheral ends of the transected L4&5 ventral roots were then placed on bipolar platinum stimulating electrodes. A pool was formed by the skin and muscle on the back and the exposed spinal region was filled with warmed (37°C) mineral oil.

Following the laminectomy and placing the L4&5 ventral roots on the stimulating electrodes, the decerebration was performed to exclude the confounding effects of anesthesia. To perform the decerebration, a transverse section was made anterior to the superior colliculus and extending ventrally to the mammillary bodies. All tissues from rostral to the section were removed. Once the decerebration was complete, anesthesia was removed from the inhaled mixture. A recovery period of 60 min was allowed after decerebration for the elimination of the effects of anesthesia from the preparation.

Static muscle contractions in the right hindlimb were then performed by the electrical stimulation of the L4&5 ventral roots (30 s, 3‐times motor threshold with a period of 0.1 ms at 40 Hz) in control rats (n = 19) and occluded rats (n = 26). To verify the exercise pressor reflex in control and occluded rats, static muscle contraction was performed on 8 control rats and 12 occluded rats. To examine the dosage effect of BAY‐87 on exercise pressor reflex in control and occluded rats, static muscle contraction was performed after the arterial injection of saline (for control and recovery) and 3 hr after the injection of BAY87 in six control rats and six occluded rats. To verify the time‐course effect of BAY‐87, static muscle contractions were performed on five control rats and eight occluded rats at 0, 30 min, 60 min, 90 min, 2 hr, 3 hr, 3.5 hr, 4 hr, 4.5 hr following 0.2 mg/kg of BAY‐87 injection.

### 2.4 Statistical analysis

Experimental data were analyzed using one‐way analysis of variance (ANOVA) or two‐way repeated measures ANOVA. As appropriate, Tukey's post hoc tests were used. All values were presented as mean ± SD. For all analyses, differences were considered significant at *p* < 0.05. All statistical analyses were performed using SPSS for Windows version 15.0.

## 3 RESULTS

### 3.1 The expression of HIF‐1α protein in the DRG

**Figure 1:**
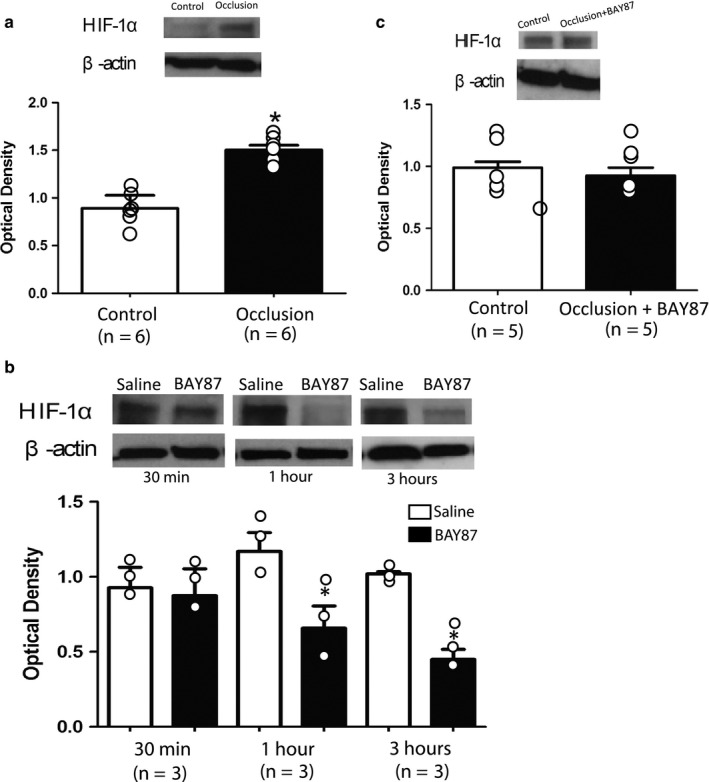
The protein levels of HIF‐1α expression in the DRG. (a) 72 hr of the femoral artery occlusion amplified HIF‐1α in the DRGs. *p < 0.05 versus control limbs. n = 6 in each group. (b) HIF‐1α in the DRGs of control limbs 30 min, 1 hr, and 3 hr after the injection of BAY87. BAY87 was injected into the right femoral artery and saline was injected into the left femoral artery for control. No significant difference was observed in HIF‐1α between the saline group and BAY87 group 30 min following their injection (*p* > 0.05). HIF‐1α was decreased 1 and 3 hr after BAY87. *p < 0.05 versus saline control. n = 3 in each group. (c) HIF‐1α in the DRGs of control limbs and occluded limbs with 3 hr of BAY87 injection. No significant difference in the levels of HIF‐1α was observed between control limbs and occluded limbs with BAY87 (*p* > 0.05 between the two groups). n = 5 in each group. Note that β‐actin was used to show equal loading of the protein in the western blot bands. Molecular weight: 93 kDa for HIF‐1α; and 42 kDa for β‐actin.

We first examined the effects of femoral occlusion on the protein levels of HIF‐1α expression in the L4‐L6 of DRGs. Figure 1a shows that HIF‐1α was increased in the DRGs of occluded limbs 72 hr after the ligation as compared with that of control limbs (optical density: 0.89 ± 0.13 in occlusion vs. 1.5 ± 0.05 in control; *p* < 0.05, n = 6 in each group).

Then, we examine if BAY87 can inhibit the levels of HIF‐1α in the DRGs of limbs without ligation. Figure 1b shows that the levels of HIF‐1α protein were not significantly changed 30 min after injection of 0.2 mg/kg of BAY87 (optical density: 0.93 ± 0.14 in saline control versus 0.87 ± 0.18 with BAY87; *p* > 0.05, n = 3 in each group). Nonetheless, BAY87 began to decrease the levels of HIF‐1α 1 hr after its injection (optical density: 1.17 ± 0.13 in saline control versus 0.67 ± 0.15 with BAY87; *p* < 0.05, n = 3 in each group) and 3 hr after its injection (optical density, 1.02 ± 0.02 in saline control versus 0.45 ± 0.07 with BAY87; *p* < 0.05, n = 3 in each group) as compared with saline controls.

We further determined the effects of BAY87 on HIF‐1α in the DRGs of occluded limbs. Figure 1c demonstrates that an increase in HIF‐1α level was attenuated in the DRGs of occluded limbs 3 hr after the injection of BAY87. Similar levels of HIF‐1α were observed in the DRGs of occluded limbs with BAY87 and in the DRGs of control limbs (optical density: 1.02 ± 0.09 with BAY87 vs. 1.06 ± 0.1 in control; *p* > 0.05, n = 5 in each group).

### 3.2 The effects of HIF‐1α inhibition on the exercise pressor reflex

**Figure 2:**
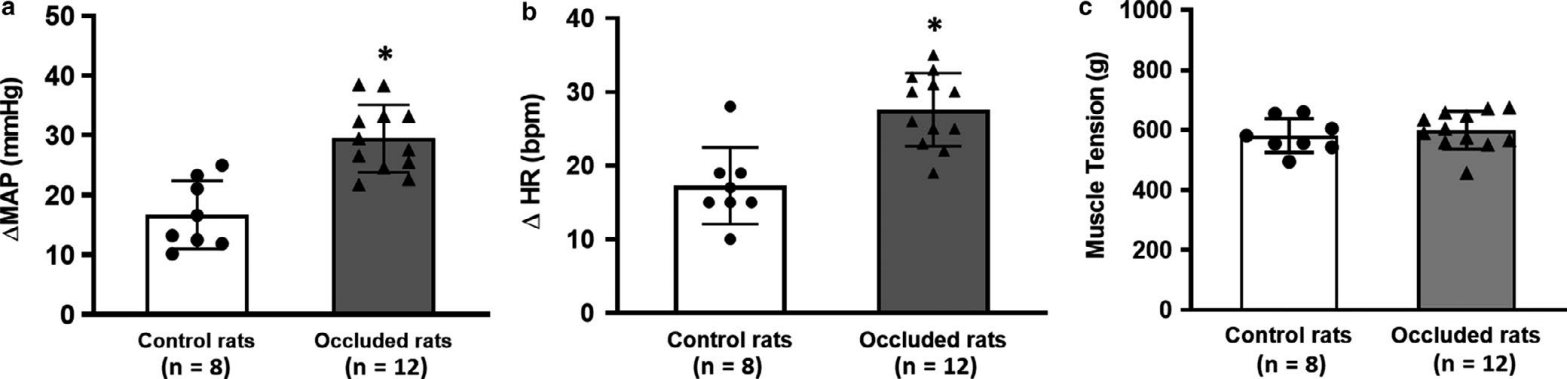
Reflex BP and HR responses induced by static muscle contraction. (a and b) Increases in MAP and HR were amplified in occluded rats as compared with control rats. *p < 0.05, control group (n = 8) versus occlusion group (n = 12). Note that no significant difference in basal MAP and HR between the two groups. Baseline BP/HR were 98 ± 8 mm Hg/385 ± 10 bpm in control rats (n = 8) and 97 ± 7 mm Hg/390 ± 12 bpm in occluded rats (n = 12; *p* > 0.05 control versus occlusion for both MAP and HR). (c) Showing no difference in peak muscle tension developed during contraction between control rats and occluded rats (*p* > 0.05 between two groups).

There were no significant differences in baseline MAP and HR between control rats (98 ± 8 mmHg/385 ± 10 bpm, n = 8) and occluded rats (97 ± 7 mmHg/390 ± 12 bpm, n = 12; *p* > 0.05 control vs. occlusion for both MAP and HR). Reflex BP and HR responses were induced by muscle contraction in control rats and occluded rats. Figure 2a,b shows that contraction increased MAP and HR; and that femoral occlusion amplified the reflex MAP and HR responses. ΔMAP and ΔHR were greater in occluded rats than these changes in control rats (*p* < 0.05, control vs. occlusion). Note that no significant difference was seen in the muscle tension development between the two groups (Figure 2c).

**Figure 3:**
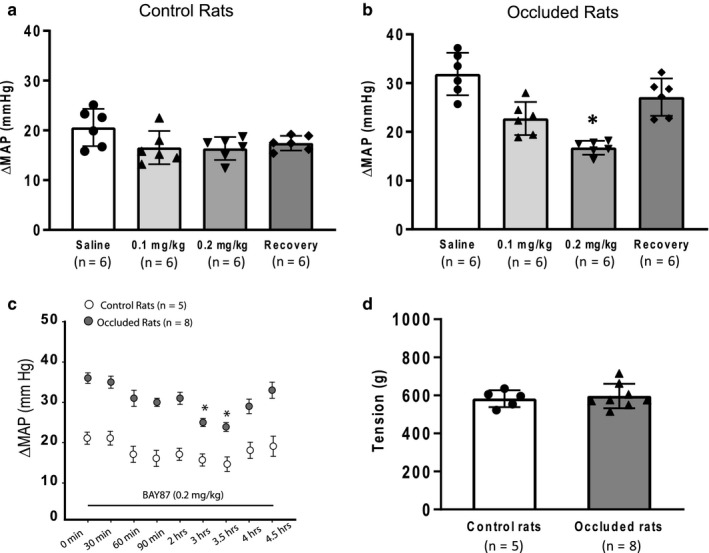
Effects of BAY87 on the reflex pressor response induced by static muscle contraction. (a and b) Showing that a greater effect was observed after 0.2 mg/kg of BAY87 was given in occluded rats (n = 6). *p < 0.05 versus saline, 0.1 mg/kg of BAY87 and recovery. (c) The time course for the effect of BAY87 on the reflex BP response induced by muscle contraction in control rats (n = 5) and occluded rats (n = 8). The maximal reduction of MAP was seen 3–4 hr after the injection of 0.2 mg/kg of BAY87. *p < 0.05 versus other time points. (d) Showing no difference in muscle tension development between control rats and occluded rats as the maximal inhibition of BAY87 was observed after its injection.

In order to verify that HIF‐1α is involved in the amplified exercise pressor reflex, BAY87 was given into the hindlimb muscles to examine its effects on reflex BP and HR responses evoked by muscle contraction. We determined dosages of BAY87 and time courses for the effects of BAY87. There was no difference in baseline MAP between the two groups: 91 ± 6 mmHg in control rats versus 89 ± 5 mmHg in occluded rats (*p* > 0.05 between two groups). Figure 3a,b show that the inhibitory effect of BAY87 on the MAP response was greater in occluded rats than that in control rats. In specific, in occluded rats, the MAP response following 0.2 mg/kg of BAY87 (16.8 ± 1.4 mmHg; *p* < 0.05 vs. all other groups) was lower than that following treatments of saline (31.9 ± 4.4 mmHg), 0.1 mg/kg of BAY87 (22.7 ± 3.4 mmHg) and recovery (27.1 ± 3.8 mmHg; Figure 3b); whereas in control rats, there was no significant difference among groups (Saline: 20.6 ± 3.7 mmHg; 0.1 mg/kg of BAY87: 16.5 ± 3.3 mmHg; 0.2 mg/kg of BAY87: 16.4 ± 2.3 mmHg; Recovery: 17.4 ± 1.5 mmHg; *p* > 0.05 among groups; Figure 3a). This result also indicated that 0.2 mg/kg of BAY87 attenuated BP response to a greater degree than 0.1 mg/kg of BAY87 in occluded rats 3 hr after they were given. The time course for the effect of 0.2 mg/kg of BAY87 on reflex BP response is presented in Figure 3c. A peak inhibitory effect of 0.2 mg/kg of BAY87 was observed 3–4 hr after its injection. No significant differences in muscle tension were observed in control rats (n = 5) and occluded rats (n = 8) as maximal effects of BAY87 were observed (Figure 3d) and at different time points after BAY87 (Table 1).

**Table 1:** Muscle tension (g) at different time points.

	0 min	30 min	60 min	90 min	2 hr	3 hr	3.5 hr	4 hr	4.5 hr
Control rats (n = 5)	565 ± 52	587 ± 62	591 ± 59	576 ± 42	589 ± 58	582 ± 45	596 ± 65	592 ± 56	573 ± 63
Occluded rats (n = 8)	546 ± 48	592 ± 54	593 ± 68	588 ± 74	595 ± 55	596 ± 64	589 ± 69	590 ± 62	596 ± 62

No significant difference in muscle tension was observed at all time points between control rats and occluded rats. Data are presented as Mean ±SD.

## 4 DISCUSSION

Femoral artery occlusion in rats has been used to study human PAD (Waters et al., 2004). Although this model does not fully exhibit all the clinical symptoms of PAD, it mimics one of the critical characteristics observed in PAD, namely, intermit claudication manifested by insufficient blood flow. In particular, in occluded rats, the blood flow limitation is observed during exercise while the resting blood flow is maintained (Waters et al., 2004). This makes it appropriate to investigate exercise physiology in PAD. Importantly, in a rat model of PAD induced by femoral artery occlusion, the SNA and BP responses are also augmented during muscle contraction and/or stimulation of muscle metabolic receptors (Li & Xing, 2012).

Thus, in the present study, HIF‐1α protein was increased in the DRGs of limbs with 72 hr femoral artery occlusion as compared with control limbs. This result is consistent with the data reported in our previous study (Gao & Li, 2013). Moreover, we demonstrated that BAY87 decreased the levels of HIF‐1α in the DRGs and a greater effect was seen 3 hr after the arterial injection of BAY87 into the hindlimb muscles. As BAY87 was injected into the arterial blood supply of the occluded limbs, HIF‐1α in the DRGs was restored to the similar levels observed in control limbs. Thus, the exercise pressor reflex was examined 3 hr after the arterial injection of BAY87 and we observed that BAY87 attenuated the amplification of the reflex BP and HR responses induced by muscle contraction in occluded rats to a greater degree as compared with control rats. It is noted that the inhibitory effects of BAY87 on the exercise pressor reflex in occluded rats became smaller with increasing recovery time (i.e., >4 hr following its injection). Taken together, we suggest that the HIF‐1α signal is a part of mechanisms leading to the exaggerated exercise pressor in experimental PAD induced by femoral artery occlusion.

DMOG, an inhibitor of prolyl hydroxylase, has been shown to stabilize or increase HIF‐1α protein and enhance the expression of downstream target genes (Jaakkola et al., 2001; Milkiewicz et al., 2004). It was reported that the inhibition of endogenous HIF‐1 inactivation by DMOG induces angiogenesis in ischemic skeletal muscles of mice (Milkiewicz et al., 2004). Accumulation of HIF‐1α induced by DMOG seems to protect tissue against myocardial ischemia injury within 3 hr (Zhao et al., 2010) and then to induce angiogenesis in ischemic skeletal muscles of mice after several days (Milkiewicz et al., 2004) or in human critical limb ischemia over 2 weeks (Ho et al., 2006). In the previous study, we observed that increases in expression of HIF‐1α protein were induced in the DRGs after the injection of DMOG into the hindlimb muscles (Gao & Li, 2013). However, BP response evoked by static muscle contraction in occluded rats was not significantly amplified by DMOG. It is speculated that the levels of HIF‐1α are greater in the DRGs after femoral artery occlusion, but an excessive increase in HIF‐1α per se is unlikely to further amplify the reflex BP response. In contrast, data of the present study indicate that blocking HIF‐1α in DRGs of occluded rats by BAY87 can attenuate the amplified BP response to muscle contraction.

Published studies have demonstrated that femoral artery occlusion induces greater expression of muscle metabolite sensitive receptors, that is, transient receptor potential vanilloid type 1 (TRPV1), purinergic P2X3, and acid‐sensing ion channels (ASICs), etc. (Li & Xing, 2012; Xing et al., 2015). Also, response amplitude of the DRG neurons with the activation of those receptors is enhanced in occluded rats (Xing et al., 2008, 2009, 2012, 2013). These alterations in expression and response of those metabolic receptors lead to augments in muscle afferent nerve‐mediated SNA and BP responses. In specific, it has been reported (Ristoiu et al., 2011) that hypoxia, one of the most dominant conditions during tissue ischemia, increased the sensitization of TRPV1 in the DRG neurons. The sensitization of TRPV1, which is relied on its phosphorylation of the serine residues and the translocation of protein kinase C (PKC)ε, was inhibited by the administration of a HIF‐1α inhibitor 2‐methoxyestradiol (Ristoiu et al., 2011). Meanwhile, the level of TRPV1 phosphorylation and the PKCε translocation were positively linked to the expression of HIF‐1α. These results suggest that HIF‐1α is involved in the process of TRPV1 sensitization via the PKCε pathway. In our present study, BAY‐87 significantly attenuated HIF‐1α expression in the DRG at 3 hr following its administration (Figure 1b,c), whereas the blood pressure response to static muscle contraction in occluded rats was suppressed at 3 and 3.5 hr after BAY87 injection (Figure 3c). It, therefore, indicates that the inhibition of HIF‐1α and subsequent alternation of the exercise pressor reflex was likely due to the reduced potentization of TRPV1 in the muscle sensory nerves.

In addition, a number of prior reports suggest that nerve growth factor (NGF) can increase expression of TRPV1, P2X, and ASIC in the DRG neurons (Anand et al., 2006; Mamet et al., 2002, 2003; Ramer et al., 2001). Interestingly, the previous work has shown that increasing HIF‐1α or inhibiting HIF‐1α prolyl hydroxylases can attenuate NGF deprivation induced‐effects on neurons, suggesting that HIF‐1α plays a regulatory role in the activity of NGF (Farinelli & Greene, 1996; Lomb et al., 2007; Xie et al., 2005). Thus, it is speculated that NGF may contribute to the effects of HIF‐1α on the augmented exercise pressor reflex by the alteration of muscle metabolic receptors in the DRG neurons after femoral artery occlusion.

In conclusion, our data show that femoral artery occlusion augments expression of HIF‐1α protein in the DRGs, and enhances BP response induced by static muscle contraction. BAY87 injected into the arterial blood supply of the hindlimb muscles decreases the protein levels of HIF‐1α in the DRGs and attenuates the amplified reflex pressor response in occluded rats during muscle contraction. Overall, it is implicated that the inhibition of HIF‐1α alleviates the exaggeration of the exercise pressor reflex in rats under ischemic circumstances of the hindlimbs as seen in PAD. With consideration of the time efficacy of BAY‐87 on HIF‐1α inhibition (3 hr following its administration) and the subsequent beneficial effect on attenuating the exaggerated exercise pressor reflex in the simulated PAD model, it shed light upon the development of the future therapeutic strategy for the clinical PAD patients.

## CONFLICTS OF INTEREST

None.
